# Factors Associated With Use of Sipuleucel-T to Treat Patients With Advanced Prostate Cancer

**DOI:** 10.1001/jamanetworkopen.2019.2589

**Published:** 2019-04-19

**Authors:** Megan E. V. Caram, Ryan Ross, Paul Lin, Bhramar Mukherjee

**Affiliations:** 1 Division of Hematology/Oncology, Department of Internal Medicine, University of Michigan Medical School, Ann Arbor; 2 Veterans Affairs (VA) Health Services Research and Development, Center for Clinical Management and Research, VA Ann Arbor Healthcare System, Ann Arbor, Michigan; 3 Institute for Healthcare Policy and Innovation, University of Michigan Medical School, Ann Arbor; 4 Department of Biostatistics, School of Public Health, University of Michigan, Ann Arbor

## Abstract

**Importance:**

Sipuleucel-T is an immunotherapy that has been approved for use in patients with asymptomatic or minimally symptomatic metastatic castration-resistant prostate cancer (mCRPC). However, sipuleucel-T may not be available to some patients because of logistics, cost, and practice structure.

**Objective:**

To identify factors associated with the adoption of sipuleucel-T across the United States.

**Design, Setting, and Participants:**

In this retrospective cohort study, patients with prostate cancer who received therapy for mCRPC (docetaxel, abiraterone acetate, enzalutamide, cabazitaxel, radium 223, or sipuleucel-T) from January 1, 2010, through June 30, 2016, were identified in a large claims database of commercially insured patients. Patients who received sipuleucel-T were compared with patients who received any of the other treatments for mCRPC but did not receive sipuleucel-T. Data were analyzed from May 3, 2018, to February 24, 2019.

**Exposures:**

Sipuleucel-T treatment.

**Main Outcomes and Measures:**

Patterns of treatment that involved the use of sipuleucel-T were elucidated, and binomial logistic regression was conducted to determine patient and physician factors that were associated with the use of sipuleucel-T and whether patients received sipuleucel-T in isolation or concurrently with other therapies.

**Results:**

Among 7272 patients who received a treatment for mCRPC, 730 (10.0%) received sipuleucel-T. Mean (SD) age of patients in the entire cohort was 73.2 (9.2) years; 6739 (92.7%) were non-Hispanic and 975 (13.4%) were black. In multivariable analysis, patients who were Hispanic (odds ratio [OR], 0.57; 95% CI, 0.38-0.86) or lived in the Pacific region (OR, 0.66; 95% CI, 0.45-0.97) had lower odds of receiving sipuleucel-T than patients who were not Hispanic or who lived in the South Atlantic region. Patients with higher incomes had greater odds of receiving sipuleucel-T than patients with incomes of less than $50 000 (OR, 1.29 [95% CI, 1.04-1.61] for $50 000-$99 000; OR, 1.43 [95% CI, 1.10-1.85] for >$99 000). Patients treated by a urologist had greater odds of receiving sipuleucel-T than patients not treated by a urologist (OR, 8.89; 95% CI, 7.10-11.11). Sixty-seven patients received concurrent therapies with sipuleucel-T, most commonly abiraterone or enzalutamide, but no factors were independently associated with patients receiving sipuleucel-T concurrent with other therapies for mCRPC.

**Conclusions and Relevance:**

In this study, 1 of 10 patients with prostate cancer who were treated for mCRPC received sipuleucel-T, with several variables associated with its use. Identifying disparities in receipt of sipuleucel-T may affect future access to this and other highly specialized cancer therapies by defining barriers to treatment that could be addressed in future studies.

## Key Points

**Question:**

How is sipuleucel-T being used to treat patients with advanced prostate cancer, and what are the factors driving its use?

**Findings:**

In this cohort study of 7272 patients treated for metastatic castration-resistant prostate cancer, 730 (10.0%) received sipuleucel-T. Factors such as a patient’s ethnicity, geographic region, income, and the specialty of the physician managing their treatment were associated with use of sipuleucel-T.

**Meaning:**

Identifying disparities in receipt of sipuleucel-T may affect future access to this and other highly specialized cancer therapies by defining barriers to treatment that could be addressed in future studies.

## Teaser

This cohort study identifies factors associated with the adoption of sipuleucel-T to treat US patients with asymptomatic or minimally symptomatic metastatic castration-resistant prostate cancer.

## Introduction

Sipuleucel-T is an autologous cellular immunotherapy product that was approved by the US Food and Drug Administration (FDA) in 2010 for use in patients with metastatic castration-resistant prostate cancer (mCRPC) who are minimally symptomatic or asymptomatic.^1^ Before approval of sipuleucel-T, the only therapy that had demonstrated a benefit in overall survival in patients with mCRPC was docetaxel.^2^ After sipuleucel-T was approved, several other therapies came into the market, including cabazitaxel in 2010,^3^ abiraterone acetate in 2011,^4^ enzalutamide in 2012,^5^ and radium 223 in 2013.^6^ Sipuleucel-T is the only immunotherapy approved for the treatment of prostate cancer and the first therapeutic vaccine approved for cancer.^7^ Approval for sipuleucel-T was based on the results of 3 trials that demonstrated an improvement in overall survival of patients compared with placebo but without improvement in time to disease progression.^1,8,9^ Sipuleucel-T is relatively well tolerated with minimal adverse effects for most patients, including fever, headaches, chills, and myalgias that are typically 1 to 2 days in duration.^10^

Despite its reported efficacy in prolonging overall survival in men with mCRPC and the minimal associated adverse effects compared with other available treatments, several additional challenges are likely to have affected the adoption of sipuleucel-T by medical practices. First, significant controversy has surrounded sipuleucel-T for more than a decade. Much of this controversy originated in 2007 when the initial application to the FDA by the manufacturer of sipuleucel-T was denied. The FDA’s Oncologist Drugs Advisory Committee had voted in favor of the efficacy and safety of sipuleucel-T in 2007, but several letters from top academic oncologists opposing the committee results came out soon after that decision, citing concerns that the trials demonstrating survival benefit were not powered for overall survival, the trials did not meet their primary end point, and balance was lacking between the treatment and control arms.^11,12^ In addition, concern about potential harms to patients in the control arm of the studies remained because overall survival of patients in the control groups was shorter than would be expected for patients with similar characteristics. A hypothesis for the discrepancy in survival of the control arm compared with historical controls is that older patients who underwent depletion of their leukocytes in the placebo arm may have been harmed by the placebo therapy,^13^ a theory that has been refuted.^14^ After an additional phase 3 study was requested by the FDA and completed, sipuleucel-T was formally approved by the FDA in 2010, with a cloud marring its entry into the market.

Second, sipuleucel-T is costly; it entered the market at a cost of approximately $100 000 for a full course of therapy^15^ and is given for 1 month with costs declared up front. This process differs from other therapies that may incur similar overall costs during the course of treatment but with costs distributed for several months with less shocking monthly price tags. Shortly after sipuleucel-T’s approval by the FDA, the Centers for Medicare & Medicaid Services (CMS) launched a National Coverage Determination inquiry process to determine whether sipuleucel-T should be reimbursed nationally given its high cost. The inquiry determined in 2011 that sipuleucel-T would be reimbursed by the CMS,^7^ but this delay may have swayed some potential early adopters of the treatment. Third, an up-front investment on the part of practices is needed to deliver sipuleucel-T, including access to and training of an apheresis center to conduct leukapheresis and transport the patient’s product to the manufacturer. In addition, abiraterone and enzalutamide were approved shortly after sipuleucel-T was approved by the FDA, and coverage was approved by the CMS. The initial delay in FDA approval, the delay for reimbursement after its approval, the investment required for practices to offer sipuleucel-T, and the availability of other nonchemotherapy treatments likely all affected early adoption of sipuleucel-T.

Previous work has demonstrated that sipuleucel-T may not be widely used compared with other available therapies for mCRPC.^16^ This study sought to investigate patient, physician, and regional factors associated with the adoption of sipuleucel-T in the context of other available therapies. We used a large national database of commercially insured patients to investigate the contemporary use of sipuleucel-T across the United States, factors associated with its use, and whether it is commonly used concurrently with other therapies in patients, which is a non–evidence-based practice. Although the efficacy and role of sipuleucel-T in the treatment of mCRPC may be controversial, the factors that may be associated with differential access to this complicated and expensive but well-tolerated therapy will be salient for the adoption of other novel and complicated therapies in this new era of immunotherapy.

## Methods

### Cohort Identification

**Figure 1.**
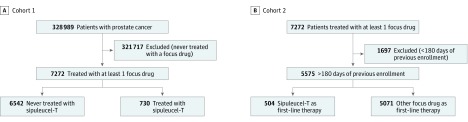
Diagram of Cohort Selections A, Cohort 1 included all patients treated with a focus drug. B, Cohort 2 included only those patients from cohort 1 who had continuous prior enrollment.

Using the deidentified Clinformatics Data Mart Database (OptumInsight) from a large US national insurer, we identified a cohort of men with prostate cancer who used sipuleucel-T or 1 of the other 5 treatments (abiraterone, enzalutamide, docetaxel, cabazitaxel, or radium 223) approved for treatment of mCRPC from January 1, 2010, through June 30, 2016 (cohort 1). Prescription records were recognized through National Drug Codes, brand names, and Healthcare Common Procedure Coding System codes and matched to respective fields within the medical claims and pharmacy claims data. Cohort 1 was used for the primary objective of determining patient and prescriber factors associated with receipt of sipuleucel-T (Figure 1).

A secondary objective was to identify how many patients received sipuleucel-T as first-line therapy and subsequent treatments. To accurately identify first-line treatment, the initial cohort 1 was restricted to patients who were continuously enrolled for 180 days before receipt of the first of the 6 treatments (cohort 2) (Figure 1). Once patients start a treatment for mCRPC, a break of 180 days between therapies is unusual. Another secondary objective was to determine whether therapies were given concurrently with sipuleucel-T and what factors were associated with concurrent use of therapies. We used a variation of cohort 1 for this objective (eFigure in the Supplement).

This study followed the Strengthening the Reporting of Observational Studies in Epidemiology (STROBE) reporting guideline for cohort studies.^17^ This study was determined to not be regulated by the University of Michigan Institutional Review Board because the research did not interact with or obtain identifiable private information about human participants.

### Variables of Interest

Because of restrictions on identification of patients inherent to the database, all data were deidentified. To determine the association of different patient variables with receipt of sipuleucel-T, we extracted sociodemographic variables, including race and ethnicity. These sociodemographic variables were determined for each patient through a demographic-based analytical model used by OptumInsight (KBM). Geographic region (New England, East North Central, West North Central, South Atlantic, East South Central, West South Central, Mountain, and Pacific) was determined by the patient’s zip code, which was encrypted so that we were only able to identify a patient’s region. New England includes Connecticut, Maine, Massachusetts, New Hampshire, Rhode Island, and Vermont. Middle Atlantic includes New Jersey, New York, and Pennsylvania. East North Central includes Illinois, Indiana, Michigan, Ohio, and Wisconsin. West North Central includes Iowa, Kansas, Minnesota, Missouri, Nebraska, North Dakota, and South Dakota. South Atlantic includes Delaware, Washington, DC, Florida, Georgia, Maryland, North Carolina, South Carolina, Virginia, and West Virginia. East South Central includes Alabama, Kentucky, Mississippi, and Tennessee. West South Central includes Arkansas, Louisiana, Oklahoma, and Texas. Mountain includes Arizona, Colorado, Idaho, Montana, Nevada, New Mexico, Utah, and Wyoming. Pacific includes Alaska, California, Hawaii, Oregon, and Washington. Thirty-five patients with unknown region in the group with no sipuleucel-T were not included in the logistic regression.

Preexisting comorbid diseases that commonly affect patients with mCRPC and are also known to be associated with treatment decisions were recorded for each patient. A comorbid disease was defined as the occurrence of at least 2 diagnosis codes for that disease (using the *International Classification of Diseases, Ninth Revision* [2008-2012] and *International Statistical Classification of Diseases and Related Health Problems, Tenth Revision* [2013-2016] codes from the Elixhauser Comorbidity Index^18^ and Clinical Classifications Software^19^) within the 2 years before receipt of the first-line treatment.

Health care professional specialty was determined through self-reported taxonomy within OptumInsight. We initially categorized the specialties as medical oncologist, urologist, radiation oncologist, or other based on the patient’s first treatment in the study time frame. Because most physicians were either medical oncologists or urologists, we condensed the categories to urologist and other. Nonphysicians who prescribe medications, such as nurse practitioners or physician assistants, were included in the other category. Details surrounding specialty identification can be found in eMethods in the Supplement.

### Statistical Analysis

Data were analyzed from May 3, 2018, to February 24, 2019. We compared the specialty of the health care professional who wrote the prescription and the demographic, economic, and clinical characteristics of patients who received sipuleucel-T with those of patients who did not by using simple binomial logistic regression. We also analyzed factors associated with whether a patient received another therapy concurrently with sipuleucel-T vs sipuleucel-T alone by using multivariable binomial logistic regression. Odds ratios (ORs) and Wald-type 95% CIs were calculated for each variable in the model. Each analysis was adjusted for all the listed variables in a multivariable analysis. All statistical analysis was performed in R (version 3.5.0).^20^

## Results

Demographics of the 7272 patients included in cohort 1 are shown in Table 1. Most of the patients were 65 years or older (6001 [82.5%]), with a mean (SD) age of 73.2 (9.2) years. Most patients were white (4764 [65.5%]) and non-Hispanic (6739 [92.7%]). A total of 975 black patients (13.4%) were included in cohort 1. Figure 1 shows the patients identified for analysis from 2010 through mid-2016 for both cohorts.

**Table 1.** Patient Characteristics and Logistic Regression of Variables Associated With Receipt of Sipuleucel-T

Characteristic	Treatment Group, No. (%) of Patients (n = 7272)^a^		Unadjusted Analysis, Odds Ratio (95% CI)^b^	Nagelkerke *R*^2^	*P* Value	Multivariate Analysis, Odds Ratio (95% CI)^c^
	Sipuleucel-T (n = 730)	No Sipuleucel-T (n = 6542)				
Age, y						
<55	22 (3.0)	199 (3.0)	1 [Reference]	0.003	.02	1 [Reference]
55-64	120 (16.4)	930 (14.2)	1.17 (0.72-1.89)			1.05 (0.63-1.73)
65-74	287 (39.3)	2321 (35.5)	1.12 (0.71-1.77)			1.01 (0.62-1.65)
≥75	301 (41.2)	3092 (47.3)	0.88 (0.56-1.39)			0.71 (0.43-1.18)
Race/ethnicity						
White	529 (72.5)	4235 (64.7)	1 [Reference]	0.007	<.001	1 [Reference]
Asian	11 (1.5)	131 (2.0)	0.67 (0.36-1.25)			0.71 (0.37-1.39)
Black	87 (11.9)	888 (13.6)	0.78 (0.62-0.96)			0.81 (0.61-1.09)
Hispanic	30 (4.1)	503 (7.7)	0.47 (0.33-0.70)			0.57 (0.38-0.86)
Unknown	73 (10.0)	785 (12.0)	0.74 (0.57-0.96)			0.85 (0.53-1.38)
Educational attainment						
No college	187 (25.6)	1982 (30.3)	1 [Reference]	0.003	.003	1 [Reference]
Some college	488 (66.8)	3948 (60.3)	1.31 (1.10-1.56)			1.11 (0.90-1.37)
Unknown	55 (7.5)	612 (9.4)	0.95 (0.70-1.30)			1.75 (0.49-6.19)
Household income, $1000						
<50	201 (27.5)	2272 (34.7)	1 [Reference]	0.11	<.001	1 [Reference]
50-99	241 (33.0)	1877 (28.7)	1.45 (1.19-1.77)			1.29 (1.04-1.61)
>99	176 (24.1)	1183 (18.1)	1.68 (1.36-2.08)			1.43 (1.10-1.85)
Unknown	112 (15.3)	1210 (18.5)	1.10 (0.81-1.48)			1.03 (0.75-1.43)
Geographic region^d^						
South Atlantic	35 (4.8)	306 (4.7)	1 [Reference]	<0.001	.96	1 [Reference]
New England	68 (9.3)	551 (8.4)	1.03 (0.70-1.50)			1.03 (0.68-1.55)
Middle Atlantic	184 (25.2)	1651 (25.2)	1.11 (0.82-1.49)			0.91 (0.65-1.26)
East North Central	106 (14.5)	993 (15.2)	0.96 (0.74-1.23)			0.89 (0.67-1.18)
East South Central	26 (3.6)	227 (3.5)	1.03 (0.67-1.59)			0.91 (0.56-1.48)
West North Central	88 (12.1)	651 (10.0)	1.21 (0.93-1.59)			1.37 (1.01-1.89)
West South Central	77 (10.5)	654 (10.0)	1.06 (0.80-1.40)			1.02 (0.74-1.42)
Mountain	98 (13.4)	611 (9.3)	1.44 (1.11-1.87)			1.27 (0.93-1.75)
Pacific	48 (6.6)	863 (13.2)	0.50 (0.36-0.69)			0.66 (0.45-0.97)
Insurance type						
HMO	172 (23.6)	2147 (32.8)	1 [Reference]	0.008	<.001	1 [Reference]
PPO	59 (8.1)	536 (8.2)	1.37 (1.01-1.87)			1.56 (1.08-2.26)
Other	499 (68.4)	3859 (58.9)	1.61 (1.35-1.93)			1.55 (1.24-1.94)
ASO						
No	600 (82.2)	5676 (86.8)	1 [Reference]	0.003	.002	1 [Reference]
Yes	130 (17.8)	866 (13.2)	1.42 (1.16-1.74)			1.04 (0.82-1.32)
Metastatic						
No	48 (6.6)	1145 (17.5)	1 [Reference]	0.02	<.001	1 [Reference]
Yes	682 (93.4)	5397 (82.5)	3.01 (2.23-4.07)			4.74 (3.26-6.89)
Comorbid conditions						
Diabetes	270 (37.0)	2180 (33.3)	1.17 (1.01-1.38)	0.001	.05	1.16 (0.97-1.39)
Hypertension	612 (83.8)	4974 (76.0)	1.63 (1.33-2.01)	0.007	<.001	1.53 (1.21-1.95)
Congestive heart failure	260 (35.6)	2272 (34.7)	0.94 (0.77-1.13)	<0.001	.50	0.84 (0.67-1.05)
Osteoporosis	146 (20.0)	1379 (21.1)	2.01 (1.63-2.48)	0.01	<.001	1.84 (1.45-2.32)
Arrhythmia	127 (17.4)	620 (9.5)	1.04 (0.89-1.22)	<0.001	.63	0.95 (0.78-1.14)
Prescriber specialty						
Other	525 (71.9)	6168 (94.3)	1 [Reference]	0.09	<.001	1 [Reference]
Urologist	205 (28.1)	374 (5.7)	6.44 (5.13-7.81)			8.89 (7.10-11.11)

Abbreviations: ASO, administrative services only (self-funded health plan); HMO, health maintenance organization; PPO, preferred provider organization.

^a^ Percentages have been rounded and may not total 100.

^b^ Among patients prescribed a treatment for metastatic castration-resistant prostate cancer, logistic regression of factors associated with the receipt of sipuleucel-T from 2010 to 2016 was conducted. Odds ratio estimates and Wald 95% CIs for each variable are reported. For each unadjusted model, Nagelkerke *R*^2^ and *P* values for a likelihood ratio test to a null model are reported.

^c^ For the multivariate model, Nagelkerke *R*^2^ = 0.24; *P* < .001.

^d^ See Methods section for states included in each region.

Overall, we found that sipuleucel-T was not very commonly used across the United States; 730 patients (10.0%) in cohort 1 who received a treatment for mCRPC received sipuleucel-T (Table 1). Of all the patients who received a treatment for mCRPC each year, the proportion who received sipuleucel-T increased from 4 of 643 (0.6%) in 2010 to 139 of 918 (15.1%) in 2012 but then steadily declined to 93 of 1077 (8.6%) by 2016 (Table 2). Most patients who received sipuleucel-T (605 [82.9%]) received all 3 recommended doses.

**Table 2.** Frequency and Proportion of Treated Patients Receiving Sipuleucel-T

Study Year	No. Receiving Any Therapy (n = 7272)^a^	No. (%) Receiving Sipuleucel-T Therapy (n = 730)
2010	643	4 (0.6)
2011	710	76 (10.7)
2012	918	139 (15.1)
2013	1110	132 (11.9)
2014	1393	147 (10.6)
2015	1421	139 (9.8)
2016	1077	93 (8.6)

^a^ Includes any treatment for metastatic castration-resistant prostate cancer (sipuleucel-T, abiraterone acetate, enzalutamide, docetaxel, cabazitaxel, or radium 223).

When restricting our cohort to patients enrolled for at least 180 days before receiving their first treatment for mCRPC (cohort 2 [n = 5575]), we found that most patients who received sipuleucel-T received it as first-line treatment (504 [69.0%]). In addition, most patients received subsequent therapies after sipuleucel-T (498 [68.2%]).

For our primary objective in the unadjusted analysis, race, educational attainment, income, insurance type, and geographic region were found to be associated with receipt of sipuleucel-T (Table 1). After adjusting for all other variables, Hispanic ethnicity (OR, 0.57; 95% CI, 0.38-0.86) and living in the Pacific region (OR, 0.66; 95% CI, 0.45-0.97) were factors that remained independently associated with lower odds of receiving sipuleucel-T. Higher household income (OR, 1.29 [95% CI, 1.04-1.61] for $50 000-$99 000; OR, 1.43 [95% CI, 1.10-1.85] for >$99 000) remained positively associated with whether a patient received sipuleucel-T, and patients with preferred provider organization (PPO) insurance had greater odds of receiving sipuleucel-T than patients with health maintenance organization (HMO) insurance (OR, 1.56; 95% CI, 1.08-2.26). Furthermore, patients treated by a urologist were several times more likely to receive sipuleucel-T at some point compared with patients treated by other specialists, such as medical oncologists (OR, 8.89; 95% CI, 7.10-11.11).

**Figure 2.**
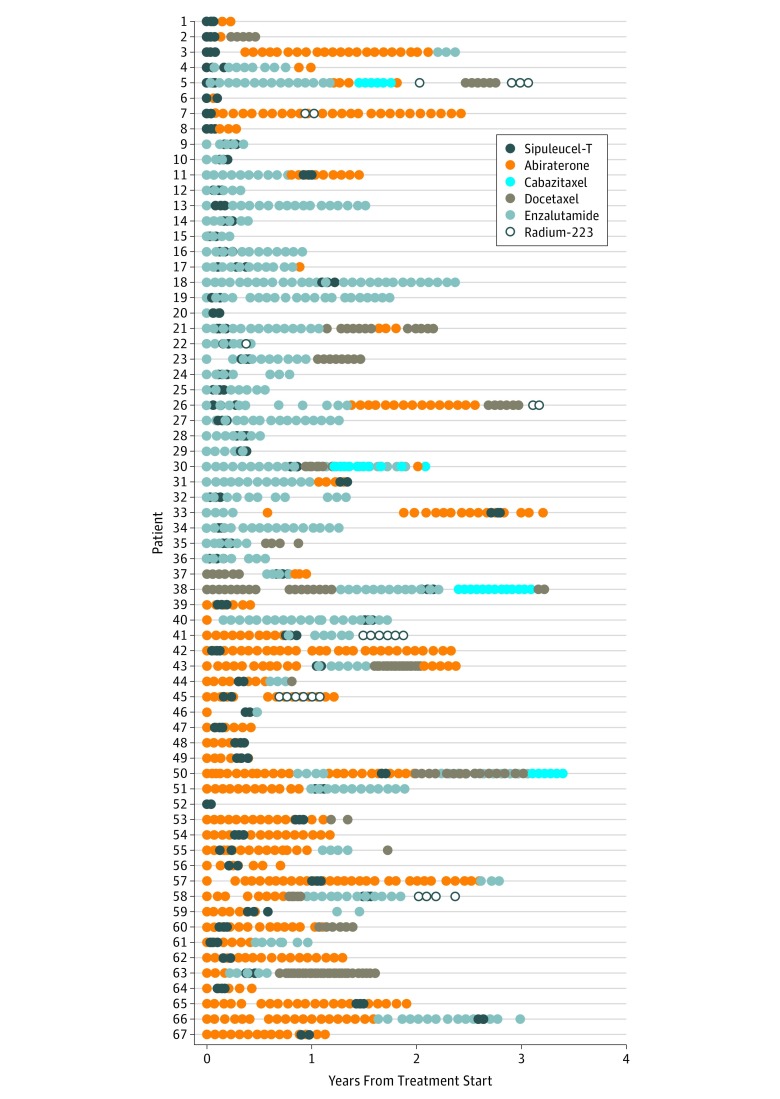
Sequence of Concurrent Sipuleucel-T Treatment Treatment courses for patients identified as receiving sipuleucel-T treatment concurrently with another therapy for metastatic castration-resistant prostate cancer. Each row represents and individual patient.

In the unrestricted cohort 1 (n = 7272), among the 730 patients who received sipuleucel-T, several (67 [9.2%]) received it concurrently with other therapies throughout the study period (most commonly abiraterone or enzalutamide), as evidenced by alternating claims. An additional 31 patients (4.3%) received another therapy within 30 days of completing their final sipuleucel-T treatment, indicating that the next therapy received was probably not started because of progressive disease but was likely planned immediately after the cessation of sipuleucel-T therapy. Figure 2 illustrates the treatment courses for patients who were identified as receiving sipuleucel-T concurrently with other therapies.^21^

After multivariable analysis (eTable in the Supplement), we found qualitative differences in the likelihood that a patient would receive sipuleucel-T concurrently with another therapy vs sipuleucel-T alone. For example, although income and educational attainment did not appear to differ between the 2 groups, Hispanic patients who received sipuleucel-T appeared to be more likely to receive sipuleucel-T concurrently with other therapies than were white patients (OR, 1.81; 95% CI, 0.64-5.15). This outcome appears to be contrary to what we found for receipt of any sipuleucel-T, as Hispanic patients were less likely to receive sipuleucel-T at any point in their disease course. Similar findings were apparent regionally, with patients in the Middle Atlantic (OR, 6.60; 95% CI, 2.71-16.05) and Pacific (OR, 3.60; 95% CI, 1.20-10.82) regions disproportionately receiving sipuleucel-T concurrently with other therapies, although these regions tended to have some of the lowest prescribing levels of sipuleucel-T compared with other regions. Nonetheless, our sample size for this regression was small, with only 67 patients receiving concurrent therapy. Therefore, although the results of this particular regression were hypothesis generating, future analyses with larger sample sizes need to be conducted.

**Figure 3.**
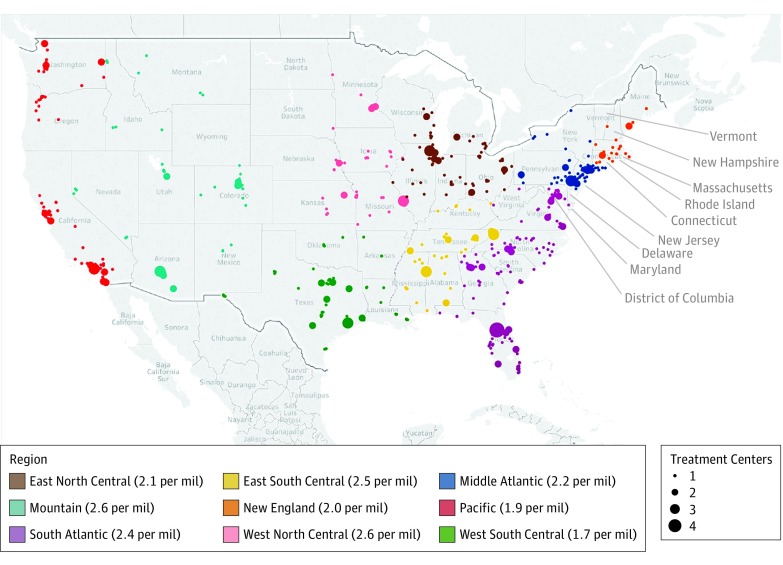
Geography of Sipuleucel-T Use Map of the United States depicts 703 centers across 635 unique zip codes that offer sipuleucel-T. Hawaii and Alaska are part of the Pacific region but are not shown here; each has 1 center. The number of treatment centers is illustrated by the size of the circle; the color of the circle illustrates the region of the country. Numbers denote the numbers of centers per 1 million (mil) people within the region. All known centers are from the website for sipuleucel-T (https://www.provenge.com/).^22^

To understand the possible contribution of geography to whether a patient received sipuleucel-T, we plotted the practices that offer sipuleucel-T^22^ on a map of the United States and calculated the number of centers per 1 million people for each region. Figure 3 illustrates the paucity of centers in the Pacific compared with other regions, such as the South Atlantic, and also demonstrates the concentration of such practices in urban areas, especially in the West North Central, Mountain, and Pacific regions of the country. Sites that offer sipuleucel-T tend to be quite sparse in areas where we found low use. The Pacific region has 1.9 centers per 1 million people, whereas the West North Central region had 2.6 centers per 1 million people. Conducting this exercise with a separate independent data source allowed us to be confident that our findings were not simply based on networks for the commercial insurer that is the source of our data but were likely based on the availability of centers.

## Discussion

In this study, sipuleucel-T was prescribed in a minority of patients who were treated for mCRPC. There may be a disparity in who has access to this treatment option. After adjusting for other variables such as age, race/ethnicity, educational attainment, and income, Hispanic patients and those living in the Pacific region were less likely to receive sipuleucel-T. Patients with a higher income were more likely to receive sipuleucel-T than were those patients with a lower income, and those with PPO insurance were more likely to receive sipuleucel-T than patients with HMO insurance. Finally, patients treated by a urologist were more likely to receive sipuleucel-T.

Several factors may have affected the adoption of sipuleucel-T soon after its approval by the FDA in 2010, including the controversy surrounding its initial denial in 2007 and the approval of other well-tolerated oral therapies shortly thereafter (abiraterone acetate was first approved for use in 2011 and enzalutamide in 2012).^12^ An additional challenge that may disproportionately affect patients with lower incomes could be the investment required by practices to arrange for patients to undergo leukapheresis in the first phase of sipuleucel-T treatment.^7^ To undergo sipuleucel-T treatment, patients must go to a leukapheresis center for harvesting of their dendritic cells, which are then shipped to the manufacturer for synthesis of the product using the patient’s autologous cells. The patient’s cells are then reinfused on 3 separate occasions at 2-week intervals.^23^ Practices that serve lower-income patients may not have the resources required to arrange and deliver such a treatment. In addition, patients who have lower incomes may not have the means to travel to seek out this treatment option even if their physicians recommend or refer them to another institution, an effect also seen in other disease states.^24,25,26,27^ The region that seemed to have a lower level of use of sipuleucel-T (Pacific) appeared to be the region in Figure 3 in which the sipuleucel-T centers were highly concentrated in urban centers, potentially explaining a disparity if patients in rural areas of the Pacific are not able to travel easily to the urban areas for treatment.

The difference could also reflect practice patterns of specialists in different states. Because most urologists do not traditionally offer chemotherapy to their patients, they may disproportionately offer sipuleucel-T to retain their patients with mCRPC rather than referring patients with mCRPC to medical oncologists. However, this practice is likely to be influenced by one’s peers and competition among other local specialists and therefore likely varies across the country and less within a state. Alternatively, patients treated by urologists may be less symptomatic, which is a recommended prerequisite for treatment with sipuleucel-T. Beyond practice resources, this therapy is also costly ($100 000 for a full course of treatment) and may have different coverage algorithms with different out-of-pocket requirements depending on a patient’s insurance plan. Patients who have PPO insurance typically pay higher premiums, which gives them more flexibility in where they go for their cancer care and may explain why having a PPO is associated with greater odds of receiving sipuleucel-T. On the other hand, patients with PPOs also tend to have higher deductibles, which may dissuade them from undergoing expensive treatments.

Tremendous controversy remains among physicians about the effectiveness of sipuleucel-T.^6^ Given the lack of prostate-specific antigen levels and radiographic responses, some critics have argued that the reason for the overall survival benefit in the absence of progression-free survival may be a result of a harmful control condition. However, this theory has been refuted,^14^ and no evidence has yet been offered to prove it. Although many of the traditional end points, such as prostate-specific antigen level and radiographic progression, have not been seen in the study of sipuleucel-T, secondary end points, such as time to first opiate use, were found to be improved in patients who were treated with sipuleucel-T compared with those not treated.^28^ An exploratory analysis we conducted among patients who received sipuleucel-T as first-line therapy vs abiraterone acetate or enzalutamide demonstrated use of a similar amount of baseline opiate equivalents in patients, suggesting patients who started sipuleucel-T therapy may not substantially differ in regard to symptoms from patients who started other therapies.

As the first therapy of its kind to enter the market, immunotherapy and vaccines for cancer were likely to be met with skepticism given their lack of apparent effects on tumors and biomarkers immediately after treatment. However, additional information is now available, and more studies are being conducted surrounding sipuleucel-T since its introduction. Sipuleucel-T was recently evaluated in the neoadjuvant setting, where it induced significant T-cell responses systemically in the patient and within the tumor itself.^29^ In the new era of immunotherapy, it is now more widely accepted that treatment responses are observed later in the course of treatment rather than within the first few weeks to months.^7^ This shifting landscape has occurred in the past few years since the approval of sipuleucel-T; thus, the rationale for the effectiveness of sipuleucel-T may gain more traction in the coming years.

### Limitations

This analysis had limitations. First, because methods to identify metastatic disease through claims are not sensitive, we included all patients with a claim for 1 of the focus treatments regardless of whether the patient had a diagnosis code for metastatic disease.^30^ Because no claim is specific to castration resistance, we were also not able to identify whether a patient had castration-resistant disease. However, we were interested in factors that drove treatment irrespective of disease status, so we were not concerned that this limitation would affect the conclusions of the study. We were also confident that the treatments we identified were used for mCRPC because all the study treatments, except docetaxel, were FDA approved only for use in mCRPC during the proposed years of analysis. Previous work^31^ has demonstrated that less than 4% of patients with prostate cancer who receive docetaxel had a co-occurring cancer for which docetaxel is approved. Another limitation to this study is that all patients had commercially available private insurance, which may cause issues with generalizability of our findings. However, because of the lack of generalizability, disparities in treatment patterns may be greater than we were able to elicit.

In addition, sociodemographic variables were generated from a composite of public records, purchase transactions, census data, and consumer surveys; thus, there is a limitation to these variables inherent to the claims database. For example, variables demonstrated to have significance in the analysis of factors associated with non–evidence-based use of sipuleucel-T (given concurrently with other therapies) were frequently the unknown variables. Thus, unknown factors may contribute to this trend; the outliers may be concentrated together regionally and potentially at the same center for which the KBM data syndicator does not have information. We may be underestimating the difference between medical oncologists and urologists because our algorithm for identifying prescriber specialty was conservative.

## Conclusions

Among patients with prostate cancer who received other therapies for mCRPC, sipuleucel-T was not commonly used. Although sipuleucel-T is the first nonchemotherapy agent approved by the FDA for mCRPC and has a favorable tolerability profile, several factors unrelated to the patient’s cancer are associated with its use, including patient income, ethnicity, and zip code region and the specialty of the prescriber. Although the optimal role for sipuleucel-T in the treatment of mCRPC is still emerging and controversy continues to surround this treatment, the story of the adoption of sipuleucel-T will be relevant for future novel therapies that may involve a similar amount of commitment on the part of practices for patients to have access to treatment. Ensuring that patients have access to effective therapies with tolerable adverse effects will be important to the cancer field moving forward. Understanding the effects that practice structure and investment as well as patient income have on treatment decisions is also important so that we can systematically address these barriers to care in future work.

## Notes

Supplement. **eMethods.** Provider Information **eFigure.** Diagram of Concurrent Cohort **eTable.** Logistic Regression of Variables Associated With Receipt of Concurrent Therapy With Sipuleucel-T (n = 67)
