# Supplementary material for: Factors Associated With Use of Sipuleucel-T to Treat Patients With Advanced Prostate Cancer
Source: JAMA Netw Open. 2019 Apr 19;2(4):e192589. doi: 10.1001/jamanetworkopen.2019.2589 (PMC6481456; doi:10.1001/jamanetworkopen.2019.2589)
Supplement: Supplement. — eMethods. Provider Information eFigure. Diagram of Concurrent Cohort eTable. Logistic Regression of Variables Associated With Receipt of Concurrent Therapy With Sipuleucel-T (n = 67) [file jamanetwopen-2-e192589-s001.pdf]

Supplementary Online Content

Caram MEV, Ross R, Lin P, Mukherjee B. Factors associated with use of sipuleucel-T to treat patients with advanced prostate cancer. *JAMA Netw Open*. 2019;2(4):e192589. doi:10.1001/jamanetworkopen.2019.2589

eMethods. Provider Information

eFigure. Diagram of Concurrent Cohort

eTable. Logistic Regression of Variables Associated With Receipt of Concurrent Therapy With Sipuleucel-T (n = 67)

This supplementary material has been provided by the authors to give readers additional information about their work.

© 2019 Caram MEV et al. *JAMA Network Open.*

eMethods. Provider Information

Provider Identification

DEA (Drug Enforcement Agency), NPI (National Provider Identifier), PROV (A unique system-generated number that identified the provider) and SERVICE_PROV (Rendering Provider on Non-Facility Claim Attending Provider on Facility Claim) codes were used to identify unique providers. Prescribing provider was either identified from pharmacy claims data or medical claims data.

DEA and NPI numbers were the variables used to link prescribing providers from pharmacy claims.

SERVICE_PROV and PROV were the variables used to link prescribing providers from medical claims.

The algorithm below outlines how providers were identified with "record" termed for describing a unique patient:

1) Pharmacy claims:
   a) For records with both NPI and DEA;
      1. Match on unique NPI and DEA;
      2. Among the remaining records, match unique NPI or unique DEA;
      3. Among the remaining records, combine partially identical columns;
   b) For records with only NPI, match on unique NPI;
   c) For records with only DEA, match on unique DEA;
2) Medical claim: match on unique SERVICE_PROV or PROV;

We used Prov_Unique, a unique system-generated number that identified the provider, to identify unique providers.

Provider Specialty Identification

Provider specialty in the Optum database is based on self-reported taxonomy.

Taxonomy codes used:

- Medical oncologist: 207RH0000X, 207RH0003X, 207RX0202X, 2080P0207X
- Urologist: 208800000X, 2088F0040X, 2088P0231X
- Radiation oncologist: 2085R0001X.
- Other: Due to the large number of taxonomies included for other individuals, facilities, and unknown, we grouped them together into a final category of "Others." It is possible that some of these providers would have been categorized as a medical oncologist, urologist, or radiation oncologist, but it was essential to the analysis that we not misclassify a provider.
  - Other individuals: 69 taxonomy codes identified other individuals that could not be assigned to either medical oncology, urology, or radiation oncology, including nurse practitioners, physician assistants, primary care providers, allopathic & osteopathic physicians from other specialties, dietary & nutritional service providers, emergency medical service providers, nursing service providers, and others. 207R00000X, 174400000X, 363A00000X, 363L00000X, 208800000X, 363AM0700X, 207P00000X, 208C00000X, 207Q00000X, 282N00000X, 363LA2200X, 207RA0201X, 207PE0004X, 363AS0400X, 2085R0203X, 163W00000X, 207L00000X, 363LF0000X, 152W00000X, 252Y00000X, 261QM0801X, 225100000X, 282NC0060X, 363LA2100X, 207RP1001X, 103T00000X, 146L00000X, 225700000X, 208D00000X, 133V00000X, 207RG0100X, 207Y00000X, 363LG0600X, 207LP2900X, 207VG0400X, 364S00000X, 2085R0202X, 390200000X, 207ZP0105X, 103G00000X, 367500000X, 2085N0904X, 207ZP0102X, 207RR0500X, 207XX0801X, 207V00000X, 101YM0800X, 207RS0012X, 207VM0101X, 2085R0001X, 208100000X, 207RG0300X, 207VX0201X, 208M00000X, 2080P0203X, 2084P0800X, 207RC0000X, 2085B0100X, 207T00000X, 225X00000X, 104100000X, 364SP0808X, 207QA0505X, 111N00000X, 208600000X, 171100000X, 2086S0127X, 1223G0001X, 207U00000X.

© 2019 Caram MEV et al. *JAMA Network Open.*

- Facility: 39 taxonomy codes identified facility, including hospitals, laboratories, agencies, and others: 261Q00000X, 282N00000X, 3336S0011X, 302F00000X, 302R00000X, 282NR1301X, 273Y00000X, 261QE0700X, 261QM1300X, 282E00000X, 251F00000X, 332B00000X, 261QU0200X, 251G00000X, 291U00000X, 315D00000X, 282NC0060X, 333600000X, 273R00000X, 3336C0003X, 332900000X, 314000000X, 284300000X, 3416L0300X, 3416A0800X, 341600000X, 261QX0200X, 3336C0002X, 251E00000X, 282NC2000X, 3336I0012X, 261QR1300X, 261QA1903X, 261QA0600X, 332BX2000X, 283X00000X, 261QS1000X, 261QR0200X, 275N00000X
- Unknown: There were also several taxonomies that could not be identified and thus we included those under unknown.

© 2019 Caram MEV et al. *JAMA Network Open.*

eFigure. Diagram of Concurrent Cohort

Figure S1: Diagram of Concurrent Cohort

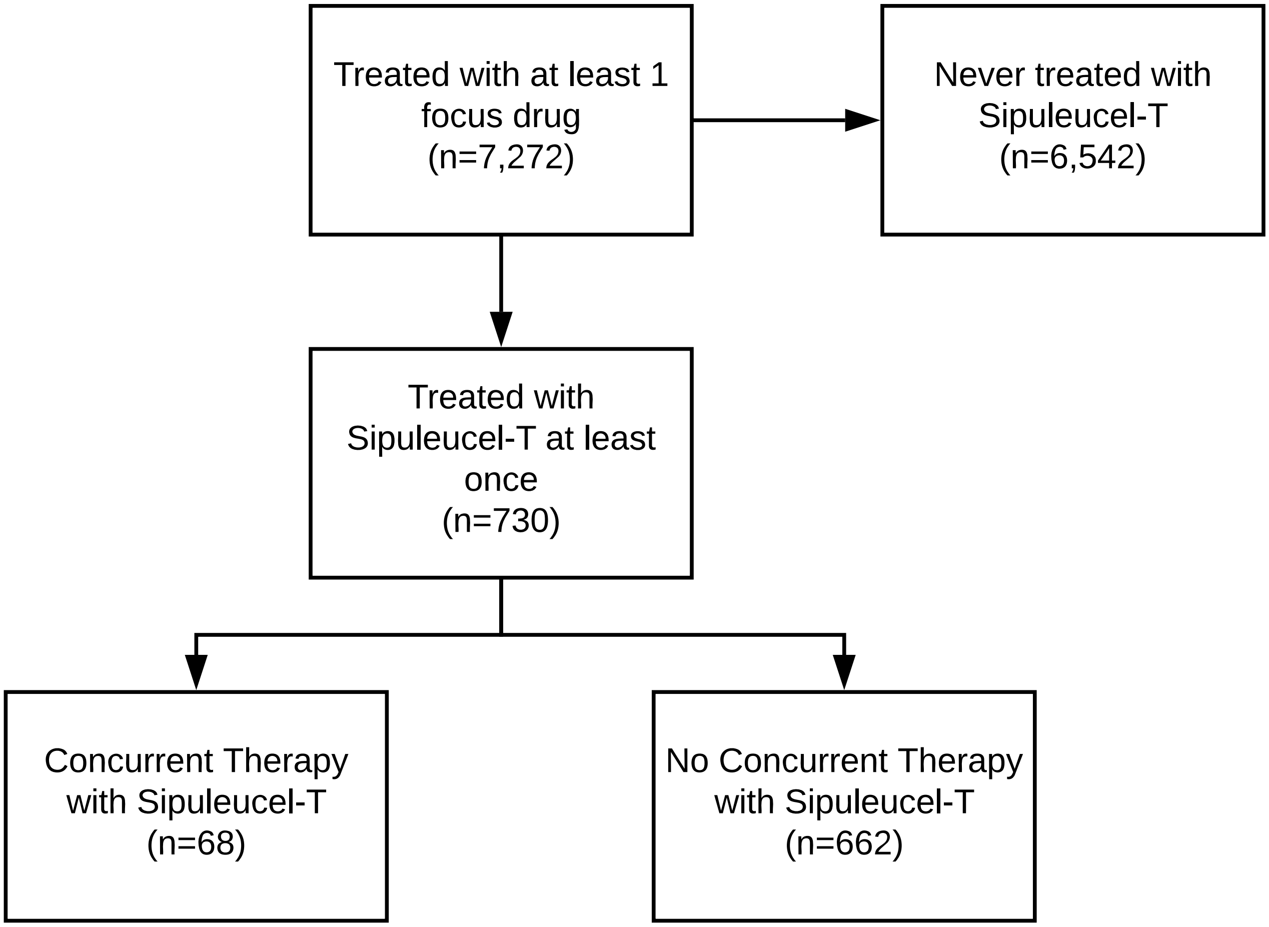

Legend: Flow diagram of the selection of the concurrent cohort selection.

© 2019 Caram MEV et al. *JAMA Network Open.*

eTable. Logistic Regression of Variables Associated With Receipt of Concurrent Therapy With Sipuleucel-T (n = 67)

	Unadjusted analysis		Multivariate analysis	
Variable	OR	95%CI	OR	95%CI
Age, y				
<55	1.00		1.00	
55-64	0.32	(0.08-0.29)	0.27	(0.07-1.11)
65-74	0.68	(0.20-2.32)	0.57	(0.18-2.04)
≥75	0.58	(0.17-1.98)	0.71	(0.10-1.44)
Race/ethnicity				
White	1.00		1.00	
Asian	1.60	(0.26-9.80)	1.19	(0.18-7.89)
Black	1.20	(0.55-2.60)	1.25	(0.55,-2.88)
Hispanic	2.41	(0.90-6.47)	1.81	(0.64-5.15)
Unknown	2.05	(1.02-4.17)	3.24	(0.99,-10.6)
Educational level				
No college	1.00		1.00	
Some college	1.17	(0.64-2.14)	0.88	(0.45-1.72)
Unknown	1.72	(0.67-4.38)	1.30	(0.03-57.81)
Household income range				
<50k	1.00		1.00	
50k-99k	1.33	(0.66-2.64)	1.38	(0.67-2.82)
>99k	1.51	(0.73-3.10)	1.78	(0.77-4.15)
Unknown	1.81	(0.71-4.63)	1.83	(0.68-4.93)
Geographic region[a]				
South Atlantic	1.00		1.00	
New England	1.25	(0.29-5.26)	1.55	(0.37-6.53)
Middle Atlantic	5.65	(2.46-12.95)	6.60	(2.71-16.05)
East North Central	0.90	(0.31-2.61)	1.07	(0.38-3.06)
East South Central	0.31	(0.02-5.81)	0.33	(0.02-5.43)
West North Central	1.53	(0.58-4.07)	1.74	(0.61-4.96)
West South Central	2.03	(0.79-5.26)	2.67	(0.74-1.42)
Mountain	1.97	(0.80-4.83)	2.44	(0.98-7.26)
Pacific	4.00	(1.55-10.33)	3.60	(1.20-10.82)
Metastatic				
No	1.00		1.00	
Yes	0.66	(0.27-1.58)	1.00	(0.31-3.31)
ASO				
No	1.00		1.00	
Yes	0.62	(0.29-1.31)	0.71	(0.32-1.57)
Comorbid Conditions				
diabetes	1.00	(0.60-1.67)	0.99	(0.57-1.73)
hypertension	1.42	(0.67-3.01)	1.30	(0.59-2.88)
CHF	0.96	(0.52-1.80)	0.99	(0.48-2.01)
osteoporosis	1.70	(0.95-3.04)	1.86	(0.98-3.51)
arrhythmia	0.75	(0.43-1.28)	0.82	(0.45-1.49)
Provider				
Other	1.00		1.00	
Urologist	1.12	(0.66-1.92)	0.93	(0.51-1.72)

© 2019 Caram MEV et al. *JAMA Network Open.*

eTable Footnote: Among patients prescribed sipuleucel-T, logistic regression of factors associated with whether a patient was prescribed another focus therapy (i.e. enzalutamide, abiraterone, docetaxel, cabazitaxel, radium-223) concurrent with sipuleucel-T. OR, odds ratio; CI, confidence interval; HMO, health maintenance organization; PPO, preferred provider organization; ASO, administrative services only (self-funded health plan); CHF, Congestive Heart Failure

[a]New England includes Connecticut, Maine, Massachusetts, New Hampshire, Rhode Island, and Vermont. Middle Atlantic includes New Jersey, New York, and Pennsylvania. East North Central includes Illinois, Indiana, Michigan, Ohio, and Wisconsin. West North Central includes Iowa, Kansas, Minnesota, Missouri, Nebraska, North Dakota, and South Dakota. South Atlantic includes Delaware, Washington, DC, Florida, Georgia, Maryland, North Carolina, South Carolina, Virginia, and West Virginia. East South Central includes Alabama, Kentucky, Mississippi, and Tennessee. West South Central includes Arkansas, Louisiana, Oklahoma, and Texas. Mountain includes Arizona, Colorado, Idaho, Montana, Nevada, New Mexico, Utah, and Wyoming. Pacific includes Alaska, California, Hawaii, Oregon, and Washington.

© 2019 Caram MEV et al. *JAMA Network Open.*
